# No Evidence for Clade I Monkeypox Virus Circulation, Belgium

**DOI:** 10.3201/eid3002.231746

**Published:** 2024-02

**Authors:** Laurens Liesenborghs, Jasmine Coppens, Christophe Van Dijck, Isabel Brosius, Irith De Baetselier, Koen Vercauteren, Marjan Van Esbroeck

**Affiliations:** Institute of Tropical Medicine, Antwerp, Belgium (L. Liesenborghs, J. Coppens, C. Van Dijck, I. Brosius, I. De Baetselier, K. Vercauteren, M. Van Esbroeck);; Katholieke Universiteit Leuven, Leuven, Belgium (L. Liesenborghs, C. Van Dijck)

**Keywords:** Mpox, monkeypox virus, MPXV, viruses, Belgium

**To the Editor:** As professionals involved in the mpox response in Belgium, we read with concern the report by Kibungu et al. on a clade I mpox outbreak linked to sexual transmission in the Democratic Republic of the Congo (DRC), in March 2023 (*1*). The authors and the World Health Organization (WHO) reported that the male index case had a sexual encounter with another man in Belgium before traveling to the DRC, where he developed symptoms the day he arrived and tested positive for monkeypox virus (MPXV) 8 days later (*2*). By that timeline, WHO suggested, the man likely was infected in Belgium. This conclusion raised concerns about clade I MPXV circulation within sexual networks in Belgium and Europe, regions highly affected by the 2022 clade IIb outbreak.

Mpox diagnoses in Belgium were mostly made by using a PCR that does not distinguish between clades (*3*). After the aforementioned reports, we retested stored samples from 296 mpox patients, 37% of all mpox patients in Belgium, by using a clade I–specific PCR (*4*). None tested positive.

In addition, from October 2022 onward, few mpox cases were reported in Belgium; none occurred in the 6 weeks before the DRC cluster started. Also, in the 9 months after the DRC outbreak, only 4 mpox cases were detected in Belgium, the earliest of which was diagnosed 12 weeks after the DRC index case (L. Liesenborghs et al., unpub. data). Moreover, during January–November 2023, we screened 2,415 men visiting our sexual health clinic using an MPXV-specific PCR as part of ongoing surveillance to detect undiagnosed or asymptomatic infections (*5*). We detected only 1 presymptomatic clade IIb MPXV infection.

On the basis of this information, we have no indications that clade I MPXV has been circulating in Belgium. However, sustained vigilance, clade differentiation, and timely outbreak investigations remain crucial to halting potential spread of clade I MPXV through sexual transmission.
